# DHA Modulates Immune Response and Mitochondrial Function of Atlantic Salmon Adipocytes after LPS Treatment

**DOI:** 10.3390/ijms21114101

**Published:** 2020-06-08

**Authors:** Marta Bou, Jacob Seilø Torgersen, Tone-Kari Knutsdatter Østbye, Bente Ruyter, Xinxia Wang, Stanko Škugor, Inger Øien Kristiansen, Marijana Todorčević

**Affiliations:** 1 Nofima (Norwegian Institute of Food, Fisheries and Aquaculture Research), 1432 Ås, Norway; marta.bou@nofima.no (M.B.); jacob.seilo.torgersen@aquagen.no (J.S.T.); Tone-Kari.Ostbye@Nofima.no (T.-K.K.Ø.); Bente.Ruyter@Nofima.no (B.R.); xinxiawang@zju.edu.cn (X.W.); Stanko_Skugor@cargill.com (S.Š.); inger.kristiansen@nofima.no (I.Ø.K.); 2 AquaGen, P.O. Box 1240, N-7462 Trondheim, Norway; 3 College of Animal Sciences, Zhejiang University, Key Laboratory of Animal Feed and Nutrition of Zhejiang Province, Hangzhou 310058, China; 4 Cargill Innovation Center, 0366 Oslo, Norway; 5 Oxford Centre for Diabetes, Endocrinology and Metabolism, Radcliffe Department of Medicine, University of Oxford, Oxford OX3 7LE, UK

**Keywords:** adipocytes, n-3 HUFAs, mitochondria, oxidative stress, antioxidant enzymes, *Salmo salar*

## Abstract

Adipocytes play a central role in overall energy homeostasis and are important contributors to the immune system. Fatty acids (FAs) act as signaling molecules capable to modulate adipocyte metabolism and functions. To identify the effects of two commonly used FAs in Atlantic salmon diets, primary adipocytes were cultured in the presence of oleic (OA) or docosahexaenoic (DHA) acid. DHA decreased adipocyte lipid droplet number and area compared to OA. The increase in lipid load in OA treated adipocytes was paralleled by an increase in iNOS activity and mitochondrial SOD2-GFP activity, which was probably directed to counteract increase in oxidative stress. Under lipopolysaccharide (LPS)-induced inflammation, DHA had a greater anti-inflammatory effect than OA, as evidenced by the higher SOD2 activity and the transcriptional regulation of antioxidant enzymes and pro- and anti-inflammatory markers. In addition, DHA maintained a healthy mitochondrial structure under induced inflammation while OA led to elongated mitochondria with a thin thread like structures in adipocytes exposed to LPS. Overall, DHA possess anti-inflammatory properties and protects Atlantic salmon against oxidative stress and limits lipid deposition. Furthermore, DHA plays a key role in protecting mitochondria shape and function.

## 1. Introduction

In commercial Atlantic salmon aquaculture, the feed in the grow-out period in sea cages usually contains high levels of lipids, which provide the main source of energy, promoting rapid growth and high feed efficiency [1,2]. However, this strategy comes at the expense of a marked increase in visceral fat deposition [3,4]. Recently, there has been much interest concerning whether similar health problems as observed for obese mammals may also occur in Atlantic salmon [5,6,7]. The traditional lipid source in fish diets comes from marine ingredients; however, there are not enough marine ingredients on the world market to cover increasing requirements from the aquaculture industry [1,2]. In order to intensify the aquaculture production further, more marine ingredients are increasingly being replaced by vegetable oils (VOs) and plant protein sources [1,8]. The continuous replacement of fish oils (FOs) with new alternative oils in aquafeeds leads to changes in the dietary fatty acid (FA) composition. Hence, alternative oils have a considerably lower level of anti-inflammatory marine n-3 highly unsaturated fatty acids (n-3 HUFAs) (eicosapentaenoic acid (EPA) and docosahexaenoic acid (DHA)), and higher levels of pro-inflammatory n-6 FAs [2,9]. The beneficial effects of EPA and DHA have been well documented in different animal and cellular models (reviewed by [10]), including Atlantic salmon [6,11]. We have previously shown that high dietary levels of EPA and DHA reduce the fat level in the visceral adipose tissue of Atlantic salmon in comparison to salmon fed oils of a vegetable origin [3]. Similarly, a long-term feeding trial showed that reduced dietary levels of EPA and DHA significantly increased the fat deposition around the viscera in Atlantic salmon [6]. In addition, oleic acid (OA) which is highly present in vegetable oils, lead to a higher lipid accumulation in Atlantic salmon adipocytes in culture compared to EPA and DHA [12,13]. As in mammals, the primary function of adipocytes and adipose tissue in Atlantic salmon is to store lipids effectively, to prevent lipotoxicity in other tissues and to sequester fat-soluble biomolecules [14,15]. Excessive fat deposition (obesity) is regarded as a low-grade inflammatory condition in mammals, since adipose tissue releases multiple pro-inflammatory cytokines and humoral factors [16,17,18]. Interactions between metabolism of lipids and immunity remain to be explored in fish. However, we have previously demonstrated that Atlantic salmon pre-adipocytes show an immune response when added the immune stimulator lipopolysaccharide (LPS) to the culture media [19]. Although not known for Atlantic salmon, in in vitro cultured human 3T3-L1 adipocytes, DHA showed anti-inflammatory effects [20]. These findings indicate the importance of increasing the knowledge of how increased fat level in combination with reduced level of marine FAs and increased FAs from plant oils, influence immune response in fish adipocytes.

The role of mitochondria in white adipose tissue (WAT) has not been studied very much in mammals or fish. However, the observations that the mitochondria number in adipose tissue decrease in obesity suggest that impaired mitochondrial activity could predispose to obesity [21]. The exact mechanisms that associate mitochondrial dysfunction with obesity remain a topic of debate [22,23]. In humans, it has been demonstrated that cellular energy metabolism is impaired in obesity, and many of the identified disturbances in energy production and use converge in the mitochondria [24]. Disruption of mitochondrial function, as seen in mammalian obesity, can increase the production of reactive oxygen species (ROS), resulting in additional cellular injury by damaging lipid membranes, nuclear and mitochondrial nucleic acids, and proteins [23]. As FAs accumulate in the cytosol, extra oxidative pathways are activated, including β-oxidation in peroxisomes and omega-oxidation in the microcosms [25], creating additional ROS and pro-inflammatory cytokines, like TNF-α, IL-10, as well as inducible nitric oxide synthase (iNOS). These pathological processes are shown to be consistent with a state of chronic systemic inflammation that is characteristic of obesity in mammals [26].

Furthermore, it has been shown in mammals that small damage to mitochondrial function may lead to reduced FA oxidation in adipocytes and thereby increased obesity [23]. In Atlantic salmon, we have seen that increased ROS damaged the mitochondrial function and β-oxidation capacity of both liver and adipose tissue [3,27], indicating that damage to mitochondrial membrane may influence the utilization of lipids for energy production also in Atlantic salmon. Although the oxidation capacity is relatively low, a minor reduction in this capacity may lead to more lipid accumulation and an increased inflammation risk. The objective of our study was to gain knowledge on how adipocytes with either high endogenous levels of OA, the major FA in rapeseed oil, or high endogenous levels of the marine FA DHA influence cellular lipid droplet formation, markers of oxidative stress and inflammation, and mitochondrial morphology and function in response to LPS treatment in Atlantic salmon.

## 2. Results

### 2.1. Characterization of Differentiated Experimental Adipocytes

**Figure 1:**
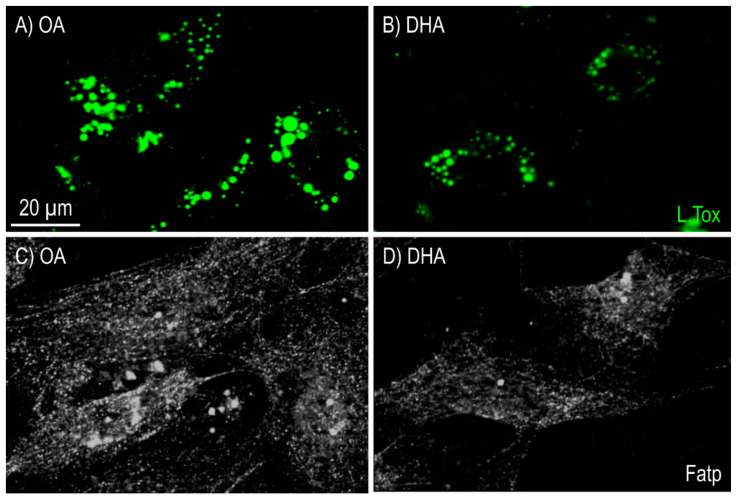
Imaging of lipid droplets by fluorescence microscopy stained with LipidTOX (green) in mature Atlantic salmon adipocytes in vitro incubated with oleic (OA) (**A**) or docosahexaenoic acid (DHA) (**B**) for 6 days. Immunofluorescence detection of fatty acid transport protein 1 (FATP1) level in mature Atlantic salmon adipocytes in vitro incubated with oleic (OA) (**C**) or docosahexaenoic acid (DHA) (**D**) for 6 days. 30 images were collected per treatment group (10 images per flask). One representative image for each treatment group is shown in this figure.

Cells in all treatment groups had a phenotype that was characteristic of mature adipocytes, with a spherical shape and filled with lipids. The lipid droplets were stained by LipidTOX and showed that cells cultivated in a medium supplemented with OA contained larger lipid droplets (Figure 1A) than cells cultivated in DHA supplemented media (Figure 1B). Immunostaining of fatty acid transport protein 1 (FATP1) revealed increased fluorescence intensity in the cells treated with OA (Figure 1C) compared to the DHA treated cells (Figure 1D), in parallel with the larger lipid droplets in the OA group (Figure 1A).

**Figure 2:**
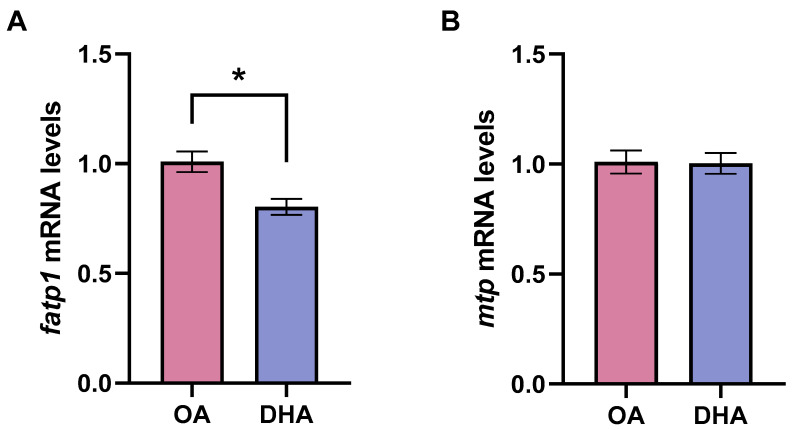
Transcript levels of fatty acid transport protein 1 (*fatp1*) (**A**) and microsomal triglyceride transfer protein (*mtp*) (**B**) in mature adipocytes incubated for 6 days with 100 µM oleic acid (OA) or 100 µM docosahexaenoic acid (DHA). Samples (*n* = 8) were analyzed with real-time qPCR; data are presented as fold change ± SEM using *ef1α* as a reference gene and the OA group was set to one (delta-delta method). Asterisks (*) indicate significant differences between conditions (*p* < 0.05; Student’s *t*-test).

Similarly, the transcript levels of *fatp1* were significantly higher in the OA group (Figure 2A) while those from microsomal triglyceride transfer protein (*mtp*) showed no difference between the different experimental groups (Figure 2B).

The endogenous FA composition of the adipocytes was significantly affected by the FA supplementation to the culture media (Table 1). Cells supplemented with OA for 6 days had a significantly higher content of OA than cells incubated with DHA (35% and 19%, respectively; *p* = 0.0003). Similarly, cells supplemented with DHA for 6 days had a significantly higher content of DHA than cells supplemented with OA (23% and 9%, respectively; *p* = 0.0003).

### 2.2. Effect of FAs on Adipocytes’ Response to LPS Treatment

#### 2.2.1. Lipid Mobilization

**Figure 3:**
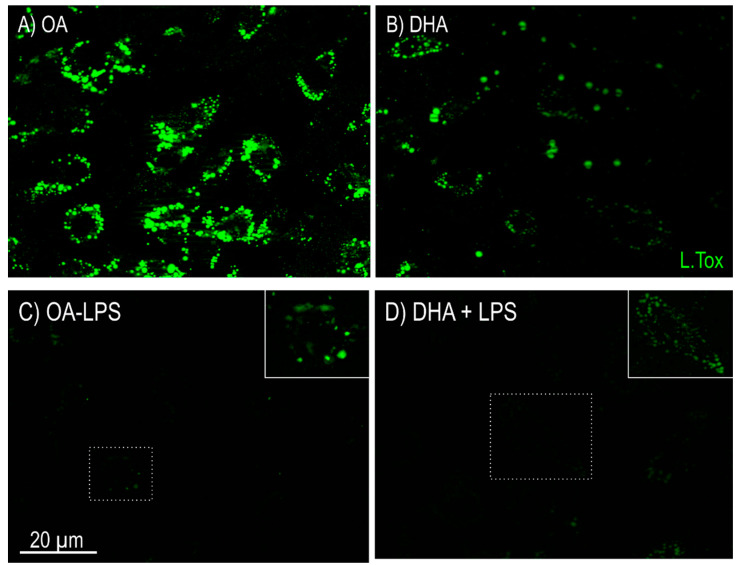
Imaging of lipid droplets by fluorescence microscopy stained with LipidTOX (green) in mature Atlantic salmon adipocytes in vitro incubated with oleic (OA) (**A**) or docosahexaenoic acid (DHA) (**B**) for 6 days. Thereafter, the OA group (**C**) and the DHA group (**D**) were exposed to lipopolysaccharide (LPS) for 20 h and imaged using the same microscopy settings. A strong lipolysis effect was observed post LPS treatment, for both OA and DHA treated cells. Boxed areas are enlarged in the upper right corners in (**C**,**D**). 30 images were collected per treatment group (10 images per flask). One representative image for each treatment group is shown in this figure.

To determine the influence of different FAs on the response to a proinflammatory stimuli, mature adipocytes were treated for 20 h with LPS in the presence of either OA or DHA. LPS exposure lead to reduced lipid accumulation in the mature adipocytes as visualized by LipidTOX staining in both OA and DHA treated cells (Figure 3A–D), indicating that LPS potentially induced lipolysis in both groups.

#### 2.2.2. Mitochondrial Morphology

**Figure 4:**
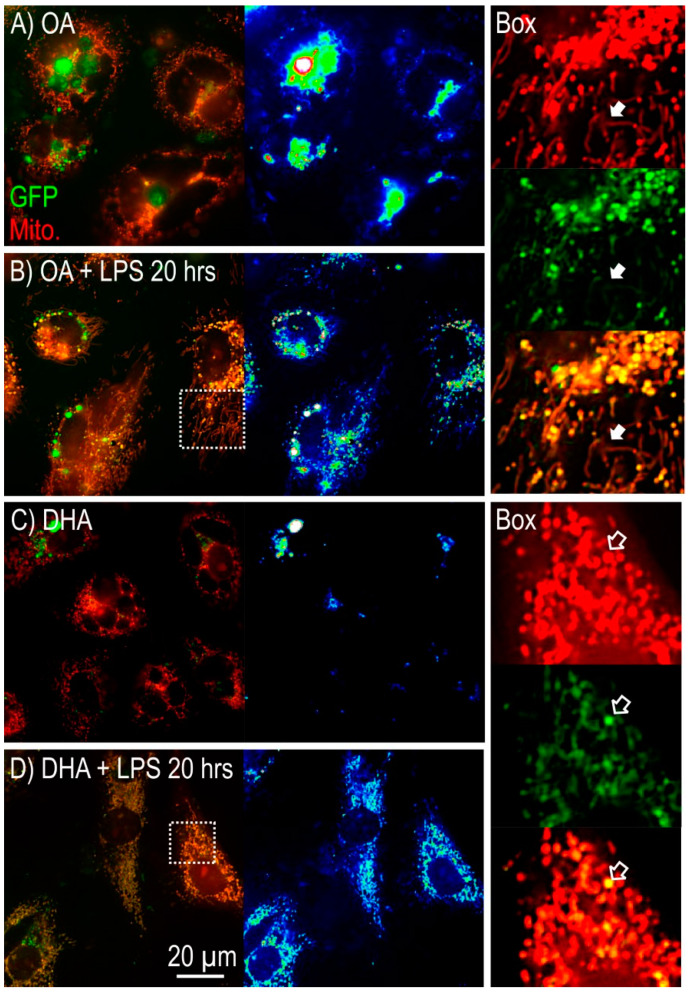
Imaging of mitochondrial morphology in mature Atlantic salmon adipocytes in vitro incubated with oleic acid (OA) (**A**) or docosahexaenoic acid (DHA) (**C**) for 6 days. Thereafter, the OA group (**B**) and the DHA group (**D**) were exposed to lipopolysaccharide (LPS) for 20 h. Mitochondria were visualized using MitoTracker (red) and a mitochondrial targeting SOD2-GFP construct (green). The panel on the right shows the area highlighted in the white dotted box for (**B**,**D**), showing the red channel, the green channel, and the merged image (colocalization shown in orange/yellow). Solid arrows point to fragmented mitochondria and open arrows point to round mitochondria.

Figure 4 shows the effect of OA and DHA on mitochondrial morphology before and after treatment with the inflammatory stressor LPS. The experimental groups were pre-transfected with the fluorescent GFP tagged superoxide dismutase SOD2-GFP vector that specifically targets the mitochondrial organelle compartment (green color). In addition, the cells were also stained with MitoTracker (red color). Colocalization of red and green fluorescence was used to achieve a more detailed image of mitochondrial morphology. In both OA and DHA treated cells, the mitochondria showed a tubular shape (Figure 4A,C). After LPS stress, the mitochondrial morphology was strongly affected by their endogenous FA composition. Cells high in OA presented a large number of elongated mitochondria with thread-like structure (Figure 4B) whereas in the DHA group LPS did not change mitochondrial morphology, which maintained a tubular shape as in the controls prior to LPS treatment (Figure 4D). Mature adipocytes treated with DHA alone showed weaker mitochondrial SOD2-GFP activity compared to OA treated cells, indicating that the higher number of lipid droplets in the latter group leads to higher expression of SOD2-GFP possibly to help in preventing mitochondrial damage due to increased oxidative stress. On the other hand, LPS treatment increased mitochondrial SOD2-GFP activity in both experimental groups, indicating that further upregulation of SOD is needed in order to prevent oxidative damage (Figure 4B,D). LPS treatment led to higher level of mitochondrial SOD2 in DHA treated group than in the OA group (opposite of the situation prior to LPS treatment).

#### 2.2.3. iNOS Activity

**Figure 5:**
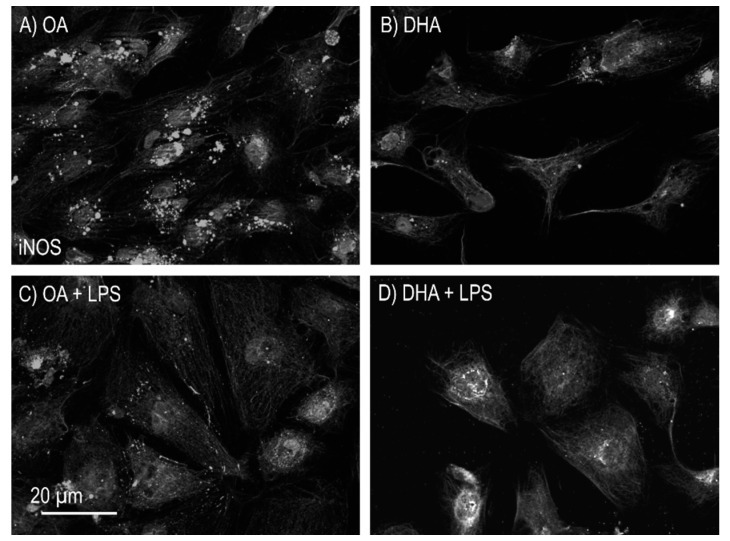
Immunofluorescence detection of inducible nitric oxide synthase (iNOS) in mature Atlantic salmon adipocytes in vitro incubated with oleic (OA) (**A**) or docosahexaenoic acid (DHA) (**B**) for 6 days. Thereafter, the OA group (**C**) and the DHA group (**D**) were exposed to lipopolysaccharide (LPS) for 20 h.

Oxidative stress responses to OA and DHA, either by themselves or in the presence of LPS in mature adipocytes were also investigated by iNOS immunofluorescence labelling. Adipocytes incubated with OA resulted in increased iNOS activity when compared to the DHA group (Figure 5). The iNOS was reduced in OA group after LPS treatment, while no mayor difference was observed for the DHA group.

#### 2.2.4. Antioxidant Enzyme Activity

**Figure 6:**
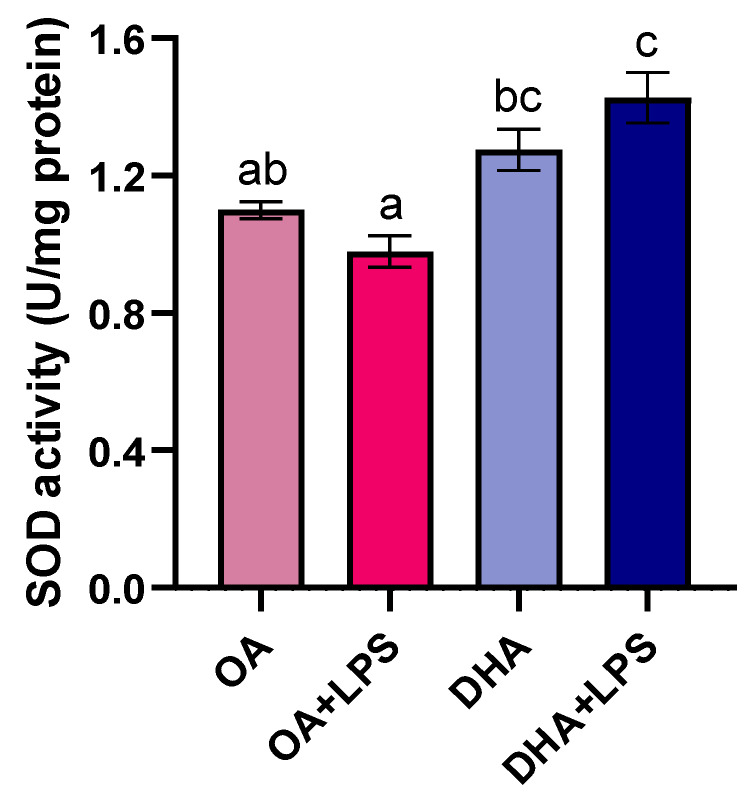
Intracellular superoxide dismutase activity (SOD) in mature adipocytes incubated for 6 days with 100 µM oleic acid (OA) or 100 µM docosahexaenoic acid (DHA) and thereafter exposed to lipopolysaccharide (LPS) for 20 h (OA+LPS and DHA+LPS, respectively). Data are presented as mean ± SEM (*n* = 4). Different letters indicate significant differences between treatments (*p* < 0.05, ANOVA followed by Tukey’s post hoc test).

After LPS treatment, the enzyme activity of SOD in the DHA group was significantly higher than in the OA group, in agreement with the findings on mitochondrial SOD2-GFP activity (Figure 6). The activity of catalase was not significantly different between the experimental groups (data not shown).

#### 2.2.5. Transcriptional Responses to FAs and LPS Exposure

**Figure 7:**
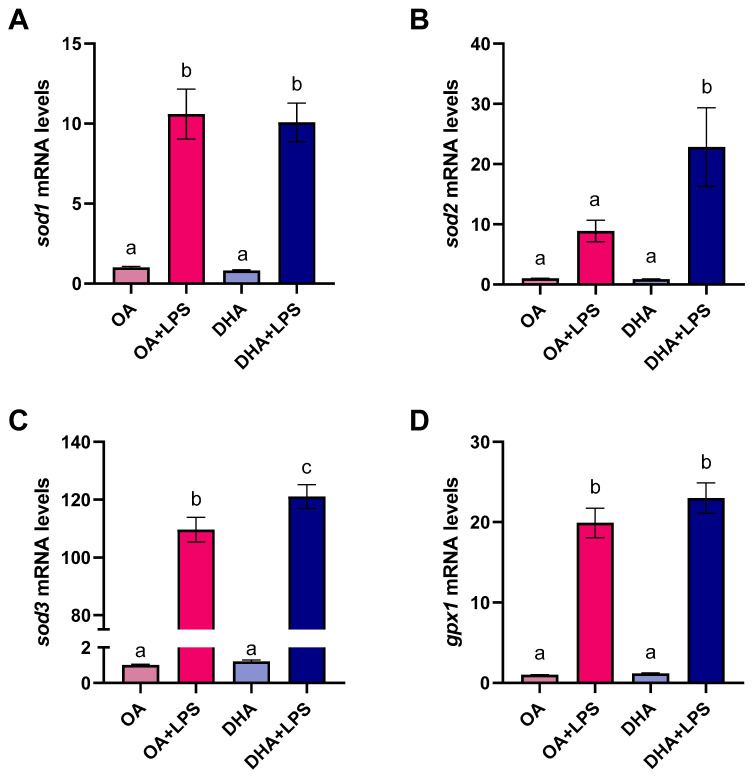
Transcript levels of superoxide dismutase 1 (cytosolic) (*sod1*) (**A**), superoxide dismutase 2 (mitochondrial) (*sod2*) (**B**), superoxide dismutase 3 (extracellular) (*sod3*) (**C**), and glutathione peroxidase 1 (*gpx1*) (**D**) in mature adipocytes incubated for 6 days with 100 µM oleic acid (OA) or 100 µM docosahexaenoic acid (DHA) and thereafter exposed to lipopolysaccharide (LPS) for 20 h (OA+LPS and DHA+LPS, respectively). Samples (*n* = 8 for OA and DHA groups and *n* = 6 for OA+LPS and DHA+LPS) were analyzed with real-time qPCR. Data are presented as fold change ± SEM using *ef1α* as a reference gene and the OA group was set to one (delta-delta method). Different letters indicate significant differences between treatments (*p* < 0.05, ANOVA followed by Tukey’s post hoc test).

The transcript levels of three isoforms of SOD; namely cytoplasmatic superoxide dismutase (*sod1*), mitochondrial superoxide dismutase (*sod2*), and extracellular superoxide dismutase (*sod3*), revealed lack of response to the FA the cells were incubated with (Figure 7A–C). However, the transcript levels of *sod1* and *sod3* were significantly increased by LPS treatment in both experimental groups (i.e., cells with high content of OA and cells with high content of DHA; Figure 7A,C). Furthermore, cells enriched in DHA subjected to LPS stimulation had a significant increase in *sod2* and *sod3* transcript levels (Figure 7B,C). These results are in agreement with the mitochondrial SOD2-GFP activity and the intracellular SOD activity described above. The transcript levels of *gpx1,* yet another important antioxidant, were not modified by the FA treatment but significantly increased in response to LPS treatment (Figure 7D).

**Figure 8:**
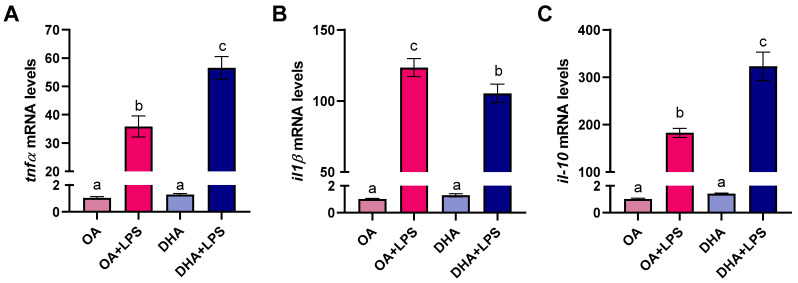
Transcript levels of tumor necrosis factor (*tnf-α*) (**A**) interleukin 1β (*il-1β*) (**B**), and interleukin 10 (*il-10*) (**C**) in mature adipocytes incubated for 6 days with 100 µM oleic acid (OA) or 100 µM docosahexaenoic acid (DHA) and thereafter exposed to lipopolysaccharide (LPS) for 20 h (OA+LPS and DHA+LPS, respectively). Samples (*n* = 8 for OA and DHA groups and *n* = 6 for OA+LPS and DHA+LPS) were analyzed with real-time qPCR. Data are presented as fold change ± SEM using *ef1α* as a reference gene and the OA group was set to one (delta-delta method). Different letters indicate significant differences between treatments (*p* < 0.05, ANOVA followed by Tukey’s post hoc test).

Gene expression analyses revealed that LPS induced pro-inflammatory cytokines like TNF-α as well as cytokine receptor IL-1β (Figure 8A,B) and the anti-inflammatory cytokine IL-10, confirming the immune role of adipocytes in Atlantic salmon. The transcript levels of these genes were not modified by the FA treatment (i.e., OA group and DHA group). However, the FAs had a significant effect when the cells were exposed to LPS. Thus, the expression of *il-1β* was significantly lower in the DHA+LPS group compared to the OA+LPS group. Opposite results were observed for *tnf-α* and *il-10*, with a significantly higher abundance of transcript levels in the DHA+LPS group compared to the OA+LPS (Figure 8 A,C).

**Figure 9:**
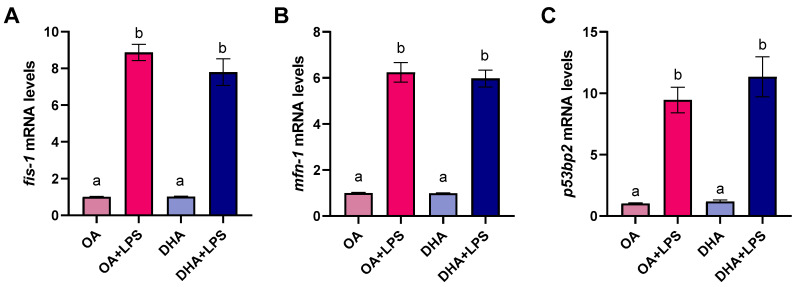
Transcript levels of mitochondrial fission protein 1 (*fis-1*) (**A**) mitofusin 1 (*mfn-1*) (**B**), and tumor protein P53 binding protein 2 (*p53bp2*) (**C**) in mature adipocytes incubated for 6 days with 100 µM oleic acid (OA) or 100 µM docosahexaenoic acid (DHA) and thereafter exposed to lipopolysaccharide (LPS) for 20 h (OA+LPS and DHA+LPS, respectively). Samples (*n* = 8 for OA and DHA groups and *n* = 6 for OA+LPS and DHA+LPS) were analyzed with real-time qPCR. Data are presented as fold change ± SEM using *ef1α* as a reference gene and the OA group was set to one (delta-delta method). Different letters indicate significant differences between treatments (*p* < 0.05, ANOVA followed by Tukey’s post hoc test).

Changes in the transcript abundance of mitochondrial fission 1 (*fis-1*) and mitofusin 1 (*mfn-1*), two genes involved in mitochondrial fission and fusion, respectively, together with changes in tumor protein P53 binding protein 2 (*p53bp2*) were assessed (Figure 9). The transcript levels of these genes were not modified by the FA the cells were incubated with (i.e., OA group and DHA group). However, LPS exposure significantly increased the mRNA levels of these three genes. Despite the lack of significance, the increase of *fis-1* and *mfn-1* was numerically higher in the OA+LPS group than in the DHA+LPS group (Figure 9A,B). On the other hand, and despite of lack significance, the increase in *p53bp2* was higher in the DHA+LPS group than in the OA+LPS group (Figure 9C).

## 3. Discussion

The primary role of white adipocytes and WAT is storage of TAG, and the secretion of adipokines comes as the secondary one reviewed in [28]. The latter is quite dependent on the first, and its activity is to a large extent affected when the storage role is compromised by cells attempting to cope with an overload of TAG (reviewed in [29]). N-3 HUFAs have been widely reported to prevent TAG overload in human adipose tissue as well as adipose tissue inflammation (reviewed in [30]). We have previously shown that Atlantic salmon adipocytes cultivated in n-3 HUFAs enriched medium had lower TAG levels than cells cultivated in a medium supplemented with OA [3,12,13,31]. In the present study, we found that DHA treatment suppressed lipid accumulation, as shown by a decrease in lipid droplet formation and lipid droplet area. Our findings are also consistent with several in vitro studies in 3T3-L1 adipocytes showing that n-3 HUFAs reduce TAG accumulation during differentiation [32,33,34]. In addition, these effects have also been reported in mammalian in vivo studies, where high-fat diets containing n-3 HUFAs limited the hypertrophy of fat depots [35,36,37,38,39,40]. The enhanced amount of lipid droplets in the OA-treated adipocytes correlated well with the higher presence of FATP1 at both the protein and transcript level, indicating its involvement in FA uptake and TAG synthesis.

The main aim of this study was to understand the effects of DHA and OA in Atlantic salmon primary adipocytes grown under standard conditions as well as under induced inflammation (LPS-treated). In mammals, apart from reducing lipid content in adipocytes, n-3 HUFAs have been reported to possess potent anti-inflammatory effects (reviewed in [10] and [41]). In the present study, we did not observe any difference in the transcriptional regulation of selected pro- and anti-inflammatory markers between the two FAs used under standard culture conditions. This is in agreement with a study performed in primary human adipocytes and adipose tissue [42]. However, in that same study the authors reported that DHA had a greater anti-inflammatory effect compared to OA under LPS-induced inflammation [42]. In line with this, DHA significantly reduced the transcript levels of the pro-inflammatory mediator *il-1β* while it increased those of the anti-inflammatory *il-10* in LPS-treated adipocytes, suggesting that DHA had greater anti-inflammatory properties than OA. Surprisingly, *tnf-α* transcripts were increased in the DHA+LPS group. Whether this increase in gene expression led to an increased secretion was not assessed in this study and a preferential post-transcriptional regulation might explain this result. Interestingly, some studies have demonstrated that the induction of adipose tissue inflammatory mediators are enhanced [43] or not affected by n-3 HUFAs [44]. These findings indicate that n-3 HUFAs can also be pro-inflammatory factors. Tai and Ding [43] reviewed that the increase in the inflammatory cytokines in response to n-3 HUFAs is probably small and may be beneficial, because they increase lipolytic activity and decrease lipogenic activity to enhance the utilization of body fat and decrease its deposition. This could also explain the observed increase in *tnf-α* transcripts in DHA treated cells under LPS-induced inflammation. However, the transcript levels do not necessarily reflect secretion, so anti-inflammatory effects need to be further studied.

We have previously shown that the intracellular redox balance is regulated during Atlantic salmon adipogenesis in order to ensure a safe lipid storage capacity [7]. On the other hand, the picture is different in overloaded adipocytes seen in obesity. Expansion of adipose tissue during obesity, as well as enlargement of adipocytes, has been linked to production of both ROS and pro-inflammatory cytokines [45,46,47]. Obese rodents and humans showed a number of elevated oxidative stress markers [48,49]. In mammals, obesity and obesity-related complications affect mitochondrial metabolism and thus favor ROS generation and the development of oxidative stress [50]. Mitochondrial ROS act as signaling molecules to link oxidative stress and inflammation [51]. Our results show that Atlantic salmon adipocytes incubated with OA had more lipids and more oxidative stress compared to the cells treated with DHA, as evidenced by an increased iNOS activity in the OA group. iNOS expression was increased in adipose tissues in genetic and dietary mice models of obesity [52]. Furthermore, Atlantic salmon adipocytes incubated with OA had a greater mitochondrial SOD2-GFP activity than adipocytes incubated with DHA. Our data suggest that this could be a defense mechanism in order to counteract the oxidative stress triggered by the higher lipid load in the OA group. On the other hand, under LPS-induced inflammation SOD2 activity was higher in the DHA enriched cells. In addition, these cells also presented a higher SOD activity and increased *sod2* and *sod3* mRNA levels, indicating that when adipocytes are subjected to inflammatory stimuli, DHA promotes antioxidant defense against oxidative stress.

In parallel with decreased SOD2-activity in the OA+LPS treated cells, morphological changes from the typical tubular shape to an unusual elongated shape with a thin thread like structures appeared in mitochondria. Changes in mitochondrial shape have been shown to affect their function [53]. In addition, elongation of mitochondria followed by cell disfunction has been reported under increased oxidative stress [54,55]. Nutrient excess has been described as one of the main factors leading to mitochondrial dysfunction and obesity-related pathologies, partly due to the production of ROS and its harmful effects [23,56]. Our data demonstrated that DHA had a positive effect in maintaining mitochondrial structure in Atlantic salmon adipocytes, even under induced inflammation. This is in agreement with the reported beneficial effects of DHA on mitochondrial function in different mammalian models (reviewed in [56]). Importantly, DHA supplementation provided protection from cardiovascular diseases [57,58] and diabetes [59] in humans, playing an important part of a healthy diet.

Taken together, the findings presented here suggest that DHA have beneficial anti-inflammatory properties and protects Atlantic salmon adipocytes against oxidative stress. The implication of DHA in maintaining mitochondrial shape and function might be one important mechanism through which this FA exerts its action.

## 4. Materials and Methods

### 4.1. Preadipocyte Isolation and Culture Conditions

Atlantic salmon (from Nofima’s sea station at Averøy, Norway) were reared on a commercial diet to an average weight of 5 kg. Randomly selected fish were transported from sea cages to land tanks with recirculating water. One at the time, the fish were anaesthetized with metacain (MS-222; 0.08 g/L, Norsk Medisinaldepot, Oslo, Norway) and killed by a sharp blow to the head. The arch bows of the gills were cut and after bleeding for a few minutes, the abdomen was cut open to expose the visible white adipose tissue surrounding the intestinal tract. The experiment was conducted according to the National Guidelines for Animal Care and Welfare of the Norwegian Ministry of Research (FOR-2015-06-18-761) and classified as not requiring a specific license (§2-f, corresponding to Directive 2010/63/EU Article 1, Section 5f), since the experimental treatments were not expected to cause any distress or discomfort for the fish, being that the fish was dead prior to adipose tissue dissection. The abdomen was cut open to expose the visceral adipose depot. Visceral adipose tissue was carefully excised, avoiding contamination with the intestinal contents. Salmon preadipocytes were isolated essentially as described by Vegusdal, et al. [60]. Briefly, the dissected fat tissue was washed with phosphate buffered saline (PBS) at pH 7.4 to carefully remove blood cells, then minced, and digested in 0.1% collagenase (type I) in L-15 (1 g tissue/5 mL L-15) at 13 °C for 1 h under shaking. All chemicals were obtained from Sigma-Aldrich Chemical Co. (St. Louis, MO, USA) unless otherwise stated.

The digested tissue suspension was subsequently filtered through 250 and 100 µm nylon filters to remove large particulate material. The resulting cell suspension was centrifuged at 700× *g* for 10 min at 10 °C. The buoyant fat layer with mature adipocytes on the top of the centrifuged tube and the digestion medium were removed by aspiration, while the preadipocytes were pelleted at the bottom. The cells obtained were washed twice, and then resuspended in growth medium containing L-15, 5% fetal bovine serum (FBS) + 5% of fish serum (FS), 2 mM L glutamine, 10 mM HEPES, and antibiotics (a mixture of penicillin, streptomycin, and amphotericin B). The adipose tissue was weighed after excision and isolated cells were seeded onto laminin coated cell culture flasks at a density of approximately 10 g tissue/25 cm^2^. The cells were cultured at 13 °C, and the media were changed every 3 days. The cells reached confluence after approximately 1 week (day 7). Confluent preadipocytes were differentiated in an initial differentiation-inducing medium that contained growth medium supplemented with 0.5 µM dexamethasone, 5 nM triiodothyronine, 12 µM isobutyl-methylxanthine, and 10 µg/mL insulin. The cells were transferred back to growth media after 48 h (day 9) and divided into two experimental groups consisting on adipocytes cultivated in growth medium supplemented with either 100 µM OA or 100 µM DHA for 6 days (OA group and DHA group, respectively). Thereafter, each experimental group was further divided into two, where some of the cells would be incubated with growth medium supplemented with 100 µg/mL lipopolysaccharide (LPS) [19] in addition to the fatty acids (OA+LPS group and DHA+LPS group). The LPS treatment lasted for 20 h. The media was replaced every 3 days. The differentiation and accumulation of lipids in the mature adipocytes were evaluated during the period of cultivation by observing the morphology of the cells.

### 4.2. Fatty Acid Composition

Three replicates from the OA and the DHA group, each replicate representing adipocytes coming from a pool of adipose tissue from an average of 5 fish, were washed twice in PBS that contained 1% albumin, washed once more with regular PBS, and harvested in 500 µL of PBS and stored at −40 °C prior to fatty acid composition of total lipids analysis. Total lipids were extracted using the method described by Folch, et al. [61] and then transmethylated overnight with 2,2-dimethoxypropane, methanolic HCl, and benzene at room temperature, as described by Mason and Waller [62] and by Hoshi, et al. [63]. The methyl esters of FAs were separated in a gas chromatograph (Hewlett Packard 6890) equipped with a split injector, using a SGE BPX70 capillary column (length 60 m, internal diameter 0.25 mm and thickness of the film 0.25 μm; SGE Analytical Science), flame ionization detector and HP Chem Station software. The carrier gas was helium, and the injector and detector temperatures were both 280 °C. The oven temperature was raised from 50 to 180 °C at the rate of 10 °C/min, and then raised to 240 °C at a rate of 0.7 °C/min. Individual fatty acid methyl esters were identified by referring to well-characterized standards. The relative quantity of each FA present was determined by measuring the area under the peak corresponding to that FA.

### 4.3. RNA Extraction and cDNA Synthesis

Eight (OA and DHA groups) or six (OA+LPS and DHA+LPS group) replicates, each replicate representing adipocytes coming from a pool of adipose tissue from an average of 5 fish were washed twice in PBS that contained 1% albumin, washed once more with regular PBS, harvested in RLT buffer containing β-mercaptorthanol, and stored at −80 °C prior to RNA extraction. Total RNA was extracted by using RNeasy^®^Mini Kit (Qiagen, Valencia, CA, USA), according to the manufacturer’s instruction. RNA was treated with RNase-free DNase I to remove any contaminating DNA. All RNA samples used in our experiments had A260/280 ratios between 1.80 and 2.30. The total RNA concentration was determined at 260 nm using spectrophotometry.

The amount of 200 ng of total RNA was reverse-transcribed into cDNA using AffinityScript QPCR cDNA Synthesis Kit (Agilent Technologies, La Jolla, CA, USA) in a 20 μL reaction system. All procedures were carried out using the following protocol: 200 ng of total RNA was used in a 20 μL reaction with a final concentration of 10 μL of cDNA Synthesis Master Mix, 3 μL of Oligo(dT) primers in a concentration of 15 ng/μL and 1 μL of AffinityScript RT/RNase Block Enzyme Mixture. The cDNA synthesis was performed with a 5 min primer incubation at 25 °C, a 45 min RT step at 42 °C, and 5 min of RT inactivation at 95 °C. The reverse transcription products (cDNA) were stored at −20 °C for qPCR of the target genes.

### 4.4. qPCR

The expression of target genes was analyzed by real-time qPCR. The PCR primers (Table 2) were designed using the Vector NTI (Invitrogen, Carlsbad, CA, USA) and synthesized by Invitrogen. Efficiency was checked from tenfold serial dilutions of cDNA for each primer pair. A 1x SYBR^®^ Green PCR Mastermix (Roche Diagnostics, Mannheim, Germany), 0.83 μM of each primer, and the cDNA template (10-fold diluted) were mixed in 12 μL volumes. PCR was performed in duplicates in 96-well optical plates on Light Cycler 480 (Roche Diagnostics, Mannheim, Germany). The specificity of PCR amplification was confirmed by melting curve analysis. Relative expression of mRNA was calculated using the 2^−ΔΔ*C*t^ method [64] using elongation factor 1α (eIF 1α) as a reference gene and the OA group was used as a control and set to one. The selected reference gene was tested for stability using the GeNorm and NormFinder. Differences between treatments were assessed with an ANOVA followed by a Tuki test (*p* < 0.05).

### 4.5. SOD2-GFP Vector

A vector aiming at monitoring oxidative stress regulation in adipocytes was designed with the endogenous SOD2 promoter and signal peptide coding sequence, to mimic endogenous transcriptional regulation and subcellular localization, respectively. Xho I/ApaI tailed PCR products were fused in frame to the 5′ end of the pZsGreen1-DR vector (Clontech, CA, USA), which features a degradable GFP variant. The vector was constructed using a PCR fragment comprising 754 bp of promoter sequence, exon I, intron I and 59 bp of exon II (GeneID: 106,571,763); primers with restriction enzyme tails; Forward: 5′CTCGAGTGTCGCCGCCATCATCCGAGA, Reverse: 5′GGGCCCAGCGAGTGCTTCCATCTGGCTGCCA). WoLF PSORT analysis of the final SOD2-GFP fusion protein predicted mainly mitochondrial localization [65].

### 4.6. Transfection

Adipocytes were transfected at 70% confluence using Lipofectamine LTX according to the manufacturer (Life Technologies™, Warrington, UK), using with 100 ng plasmid DNA, 0.38 µL of lipofectamine LTX and 0.15 µL PLUS reagent per mL of L15 media.

### 4.7. Immunofluorescence and Staining

Visualization of mitochondria and lipid droplets in live cells was achieved with MitoTracker Red and HCS LipidTOX™ Green Neutral Lipid Stain, respectively (Life Technologies). Imaging of stained mitochondria and GFP activity was carried out on adipocytes 6 days after treatment with DHA or OA and the effect of bacterial infection was monitored 20 h after addition of LPS. Immunofluorescence was carried out on PFA fixed (4% paraformaldehyde in 1 × PBS) and saponin (0.2% saponin in 1 × PBS) permeabilized cells. FATP1 mediated uptake of fatty acids was investigated using a mouse monoclonal antibody (Diluted 100× as described in Sanchez-Gurmaches, et al. [66], R&D Systems, MN, USA) and oxidative stress was studied using a rabbit polyclonal antibody against iNOS (Diluted 200×, as described in Ebbesson, et al. [67], Thermo Scientific, IL, USA). Micrographs were captured and analysed using a Zeiss Axiovision Z1 microscope and Zeiss Axiovision software, respectively (Carl Zeiss Microimaging GmbH, Göttingen, Germany).

### 4.8. Superoxide Dismutase (SOD) Assay

A commercially available kit was used to assay superoxide dismutase (SOD) activity (Cayman Chemical Company, Ann Arbor, MI, USA). The kit utilizes a tetrazolium salt for detection of superoxide radicals generated by xanthine oxidase and hypoxanthine. One SOD unit was defined as the amount of enzyme needed to exhibit 50% dismutation of the superoxide radical. The adipocytes were washed twice with 1.0% of albumin in PBS. The cells were harvested in 1 mL of PBS and centrifuged at 100× *g* for 5 min at 4 °C. Pelleted cells were stored in 100 μL PBS at −80 °C until analysis. Prior analysis, PBS covering the cells was replaced by 500 μL of PBS. The assay mix consisted of 200 μL of diluted radical detector, 10 μL of sample or standard and 20 μL of diluted xanthine oxidase. After incubation on a shaker for 20 min at room temperature, the absorbance was measured at 450 nm in a Victor 3 microplate reader (PerkinElmer Life and Analytical Sciences, Shelton, CT, USA).

### 4.9. Catalase Assay

The presence of peroxisomes was assessed by the activity of catalase, using a method based on that of Baudhuin, et al. [68]. The substrate hydrogen peroxide (Riedel-de Haen, Seelze, Germany), which is produced in peroxisomes, is broken down by catalase to oxygen and water. This reaction is stopped by the addition of a saturated solution (0.45%) of titanium oxysulphate (Riedel-de Haen) in 2 N sulphuric acid (Merck, Darmstadt, Germany). Titanium oxysulphate reacts with the remaining hydrogen peroxide to give a yellow solution of peroxy titaniumsulphate. The amount of this product was measured spectrophotometrically at 405 nm in a Victor 3 1420 Multilabel counter spectrophotometer (Perkin Elmer, Norwalk, CT, USA).

### 4.10. Statistics

Wells were used as experimental units. The normality of the data was tested using the Shapiro–Wilk test. Data were analyzed by one-way analysis of variance (ANOVA) followed by the Tukey’s honest significant difference post hoc test to detect differences between the experimental groups. If only two experimental groups were compared, Student’s *t*-test was applied. Differences were considered statistically significant at *p* < 0.05. Values are shown as means ± standard error of the mean (SEM). All statistical analyses were conducted using the software JMP^®^ version 13.1.0 (SAS Institute Inc., Cary, NC, USA, 1989-2007) and GraphPad Prism 6 (La Jolla, CA, USA, www.graphpad.com).

## Figures and Tables

**Table 1:** Fatty acid composition (% of total) in mature Atlantic salmon adipocytes. Differentiated cells at day 9 were incubated with oleic (OA) or docosahexaenoic acid (DHA) for 6 days (mean ± SEM; *n* = 3).

	OA	DHA	*p*
14:0	1.0 ± 0.3	1.1 ± 0.3	0.803
16:0	10.2 ± 0.8	11.3 ± 0.2	0.231
18:0	7.4 ± 0.2 ^a^	8.2 ± 0.2 ^b^	0.032
ƩSFA ^1^	20.2 ± 0.9	21.4 ± 0.6	0.318
16:1n-7	1.2 ± 0.5	1.7 ± 0.3	0.457
16:1n-9	0.4 ± 0.2	0.6 ± 0.1	0.338
18:1n-7	2.3 ± 0.1	2.5 ± 0.1	0.208
18:1n-9	34.8 ± 1.1 ^a^	19.2 ± 0.6 ^b^	0.0003
18:1n-11	0.8 ± 0.1	0.7 ± 0.1	0.728
20:1n-9	2.5 ± 0.3	2.2 ± 0.1	0.5523
22:1n-11	1.0 ± 0.2	1.2 ± 0.1	0.349
ƩMUFA ^2^	45.4 ± 1.4 ^a^	30.7 ± 0.7 ^b^	0.0008
18:2n-6	3.9 ± 0.1	4.6 ± 0.2	0.035
20:2n-6	0.6 ± 0.0	0.4 ± 0.2	0.670
20:4n-6	5.3 ± 0.1	4.9 ± 0.4	0.412
Ʃn-6 ^3^	11.1 ± 0.6	11.1 ± 0.7	0.961
18:3n-3	1.3 ± 0.2	1.5 ± 0.3	0.680
20:5n-3	4.2 ± 0.3	4.3 ± 0.6	0.923
22:5n-3	2.9 ± 0.1	2.8 ± 0.2	0.576
22:6n-3	9.0 ± 0.6 ^a^	23.2 ± 1.0 ^b^	0.0003
Ʃn-3 ^4^	18.0 ± 0.7 ^a^	32.2 ± 1.1 ^b^	0.0003
PUFA ^5^	29.9 ± 0.7 ^a^	44.8 ± 1.2 ^b^	0.0005

^a,b^ Mean values within a row with unlike superscript letters were significantly different (*p* < 0.05; Student’s *t*-test). ^1^ Includes 15:0, 17:0, 20:0, and 22:0. ^2^ Includes 14:1n-5, 15:1, 16:1n-5, 17:1n-7, 20:1n-11, 22:1n-9, and 24:1n-9. ^3^ Includes 16:2n-6, 20:3n-6, and 22:4n-6. ^4^ Includes 16:2n-3 and 20:4n-3. ^5^ Includes 16:3n-4.

**Table 2:** Atlantic salmon primer sequences used for real-time PCR.

Gene	Accession No.	Direction	Primer Sequence 5′→3′
*ef1α*	AF321836	Forward	CACCACCGGCCATCTGATCTACAA
		Reverse	TCAGCAGCCTCCTTCTGAACTTC
*etif3*	DW542195	Forward	CAGGATGTTGTTGCTGGATGGG
		Reverse	ACCCAACTGGGCAGGTCAAGA
*β-actin*	AF012125	Forward	ACATCAAGGAGAAGCTGTGC
		Reverse	GACAACGGAACCTCTCGTTA
*fatp1*	CA373015	Forward	TGGGAGCTTGTGGGTTCAA
		Reverse	ACTTTCATGAGGCGGATTGG
*mtp*	CA042356.1	Forward	CAAAGACCAGCGTCAACAACAA
		Reverse	CGCCTCTGTCTCAAAGCTCACT
*sod1*	BT057716	Forward	TTCTGTTGTACGCTGTCCCAAAAGC
		Reverse	GCAGCTTGGTACGCAAAGTGAACA
*sod2*	BT060259	Forward	TTCCGAGTGTGCCCAGTCTTGT
		Reverse	ACAGGGAGGTGAAAGTGCATGGT
*sod3*	BT046917	Forward	TCATCGACAGCAGAGAAGAAGGGGA
		Reverse	CTGCGATGAAAGGTGGTGAGCG
*gpx1*	CA345853	Forward	CCTTCCAGTACCTGGAGTTGAATGC
		Reverse	CTCATGATTGTCTCCTGGCTCCTGT
*tnf* *α*	NM_001123589	Forward	AGGTTGGCTATGGAGGCTGT
		Reverse	TCTGCTTCAATGTATGGTGGG
*il1* *β*	CA377361	Forward	GTATCCCATCACCCCATCAC
		Reverse	TTGAGCAGGTCCTTGTCCTT
*il10*	EF165028	Forward	ATGAGGCTAATGACGAGCTGGAGA
		Reverse	GGTGTAGAATGCCTTCGTCCAACA
*mfn1*	BT072406	Forward	AGTGTGTCCAGTCTTCCGCACA
		Reverse	ACAGGCTACAGCACCCAACCTT
*fis1*	BT072691	Forward	CCCCAGGGGGCATCCTGTCTTA
		Reverse	TTGCAGCTGGCCGATCTAGCG
*p53bp2*	XM014195885.1	Forward	TTTTCCAGGCATCACCAATGAC
		Reverse	CCAGATCAGCCATGATAGCGT

Elongation factor 1A (ef1α), eukaryotic translation initiation factor 3 (*etif3*), fatty acid transport protein 1 (*fatp1*), microsomal triglyceride transfer protein (*mtp*), superoxide dismutase 1 (cytosolic) (*sod1*), superoxide dismutase 2 (mitochondrial) (*sod2*), superoxide dismutase 3 (extracellular) (*sod3*), glutathione peroxidase 1 (*gpx1*), tumor necrosis factor (*tnf-α*), interleukin 1β (*il-1β*), interleukin 10 (*il-10*), mitofusin 1 (*mfn-1*), mitochondrial fission 1 protein (*fis-1*), tumor protein P53 binding protein 2 (*p53bp2*).

## Abbreviations

DHA	Docosahexaenoic acid
OA	Oleic acid
HUFAs	Highly unsaturated fatty acids
FAs	Fatty acids
LPS	Lipopolysaccharides
